# Comparative analysis of post-operative rehabilitation approaches for medial patellar luxation in small-breed dogs

**DOI:** 10.14202/vetworld.2024.550-557

**Published:** 2024-03-07

**Authors:** Ekkapol Akaraphutiporn, Irin Kwananocha, Chularach Meechai, Pijitra Suksomboonwong, Suchanun Aramsriprasert, Ornjira Meethong, Arttapol Triampitak, Chalika Wangdee

**Affiliations:** 1 Department of Veterinary Surgery, Faculty of Veterinary Science, Chulalongkorn University, Bangkok, 10330, Thailand; 2 Research and Academic Service, Faculty of Veterinary Medicine, Kasetsart University, Bangkok, 10900, Thailand; 3 Veterinary Clinical Stem Cells and Bioengineering Research Unit, Chulalongkorn University, Bangkok, 10330, Thailand

**Keywords:** electrical stimulation, light amplification by stimulated emission of radiation therapy, medial patellar luxation, post-operative management, rehabilitation

## Abstract

**Background and Aim:**

Electrical stimulation (ES) and light amplification by stimulated emission of radiation (LASER) therapy are frequently used in post-operative rehabilitation; however, there is currently insufficient research comparing their effectiveness. This study aimed to assess the effectiveness of post-operative rehabilitation following medial patellar luxation (MPL) surgical correction by comparing ES and LASER therapy when combined with exercise. This was compared with a control group that consisted solely of post-operative home exercise implemented by the owner.

**Materials and Methods:**

We conducted a prospective clinical trial on dogs that had undergone surgical treatment for MPL. The dogs were categorized into the following three groups: The control group, which did not participate in any post-operative rehabilitation program; the ES group, which received post-operative rehabilitation involving ES therapy; and the LASER group, which underwent post-operative rehabilitation featuring LASER therapy.

**Results:**

There were no significant differences among the groups regarding the evaluation parameters, including lameness score, pain score, thigh muscle circumference, and range of motion. Although there may have been a difference in pain score in some groups, it could be attributed to the pre-operative condition of patients. These results aligned with the owner questionnaires’ canine brief pain inventory assessments, showing no significant differences between treatment groups.

**Conclusion:**

Post-operative rehabilitation for MPL correction may enhance limb usage, joint function, muscle mass, and pain relief. However, the duration and level of post-operative pain may influence the necessity for rehabilitation. In addition, ES and LASER therapy offer similar pain-relieving effects after MPL surgery; therefore, the choice between these methods depends on the availability of equipment and veterinarian preferences.

## Introduction

Patellar luxation is a common cause of lameness in small-breed dogs. Although it can occur in medial, lateral, or bidirectional directions, most cases involve medial luxation, particularly in small-breed dogs such as Chihuahua, Yorkshire terriers, and Pomeranians [1, 2]. Medial patellar luxation (MPL) is associated with various hindlimb abnormalities involving the hip joint, femur, and tibia. Although MPL is not typically life-threatening, it can significantly impact a dog’s quality of life (QOL), particularly when classified as high-grade [1, 3, 4]. Clinical signs may vary from mild lameness and pain to complete non-weight-bearing lameness. In addition, MPL may contribute to the rapid development of osteoarthritis and other stifle injuries, such as cranial cruciate ligament rupture (CrCLR) [5, 6]. Therefore, MPL treatment is often recommended, especially in young dogs, to prevent future complications [1, 2, 7].

Treatment options include conservative management and surgical intervention depending on the severity of MPL grading and overall condition of the dog [1, 2, 4, 7]. Conservative treatment strategies may include the administration of anti-inflammatory and pain-relieving medications, exercises, weight management, dietary supplements, and rehabilitation. However, conservative management mainly aims to reduce symptoms and enhance the dog’s QOL, but it may not address the luxation of the patella itself. Surgical intervention typically proves more beneficial than conservative management for dogs with a higher MPL grading, persistent lameness, or significant pain [7, 8]. Common surgical procedures involve techniques to deepen the femoral trochlear groove, such as trochlear block recession (TBR) or trochlear wedge recession, and tibial tuberosity transposition (TTT), to realign the quadriceps mechanism. Soft-tissue techniques, such as lateral imbrication and medial desmotomy, are also frequently used [1, 2, 6]. Although surgical interventions usually result in favorable long-term outcomes, some dogs may experience post-operative lameness. This may be associated with post-operative pain and inflammation, which may lead to muscle atrophy and reduced joint function. Therefore, post-operative rehabilitation may emerge as a critical component of care that helps to restore range of motion (ROM), enhance joint function, and prevent muscle atrophy [8–10].

Canine rehabilitation in veterinary medicine is gaining popularity. Non-invasive treatments include cold compression, passive ROM (PROM) exercises, standing exercises, leash walking, underwater treadmill sessions, electrical stimulation (ES), and light amplification by stimulated emission of radiation (LASER) therapy [8, 9, 11, 12]. For various indications, these therapies reduce pain and inflammation, improve ROM, and promote physical recovery while minimizing complications following surgery. ES and LASER therapy offer effective, low-risk, and safe procedures for promoting healing and pain alleviation in various clinical applications [9, 13–15]. ES consists of multiple types, characterized by different waveforms, amplitudes, and frequencies, such as electrical muscle stimulation, neuromuscular ES, functional ES, transcutaneous electrical nerve stimulation, Russian current, and interferential current (IFC), each with different therapeutic purposes [16, 17]. IFC is a specific ES modality that has gained prominence in canine rehabilitation due to its effectiveness in pain control. It delivers a high carrier frequency (approximately 4000 Hz) with amplitude modulation at low frequencies (0–250 Hz). The advantages of IFC include reduced skin impedance and amplitude-modulated frequency (AMF) parameter that penetrates deep into the treatment area. Various physiological mechanisms have been proposed to explain the analgesic effects of IFC, such as gate control theory, increased circulation, descending pain suppression, and nerve conduction block [15–17]. Similarly, LASER therapy has proven to be effective in rehabilitation. Photobiomodulation is an effective non-thermal interaction of monochromatic radiation with target tissues. LASER therapy modulates cellular functions, accelerates wound and joint healing, promotes muscle regeneration, and effectively manages acute and chronic pain, edema, neurological conditions, and post-operative care [14, 16, 18, 19].

Both ES and LASER therapy are used for pain relief. However, a comparative study assessing their effectiveness in post-operative rehabilitation is currently lacking. This study’s objective was to evaluate the effectiveness of post-operative rehabilitation overseen by a veterinarian following MPL surgical correction in small-breed dogs by comparing ES and LASER therapy when combined with exercise. This was compared with a control group that consisted solely of post-operative home exercise implemented by the owner. We hypothesized that there would be no significant differences between ES and LASER therapy and that both therapies would provide more benefits than home exercise alone.

## Materials and Methods

### Ethical approval

This study was approved by the Institutional Animal Care and Use Committee of the Faculty of Veterinary Science, Chulalongkorn University (No. 2031032).

### Study period and location

This prospective clinical trial was conducted between 2019 and 2022 at the Small Animal Hospital, Faculty of Veterinary Science, Chulalongkorn University, Thailand.

### Study design and data collection

This study involved 26 client-owned dogs, representing 31 stifles, all of whom underwent surgical treatment for MPL. Informed written consent was obtained from the owners before participation. The dogs were subsequently allocated into the following three groups: The control group, which did not undergo any post-operative rehabilitation program; the ES group, which received post-operative rehabilitation with ES; and the LASER group, which underwent post-operative rehabilitation with LASER therapy. The choice of post-operative rehabilitation therapy, either ES or LASER therapy, was determined based on the owner’s decision, whereas the selection between ES and LASER therapy for the rehabilitation group was made randomly.

### Animals

All dogs in this study underwent complete physical examinations and hematological profiling, which had to fall within the normal range. Inclusion criteria were small-breed dogs weighing <10 kg, regardless of the grade of MPL, who underwent surgical treatment. Dogs with systemic health issues, neurological problems, skeletal deformities, and other abnormal stifle conditions such as CrCLR were excluded from the study. The dogs were followed up for up to 12 weeks post-operatively to assess the outcome.

### Surgical procedures

The dogs in this study underwent anesthesia according to their individual needs. Following anesthesia, the patients were positioned in dorsal recumbency and prepared aseptically. Arthrotomy was performed using a lateral parapatellar approach, and specific surgical techniques were chosen on the basis of the condition of the stifle joint and the surgeon’s preferences. Bone reconstruction techniques included TBR and TTT, whereas soft-tissue reconstruction techniques included lateral imbrication and medial desmotomy. All dogs received antibiotic treatment (cephalexin 25 mg/kg, twice daily, for 7 days) and non-steroidal anti-inflammatory drugs (NSAIDs, carprofen 2.2 mg/kg, twice daily, for 10 days) post-operatively. Wound care was adapted to suit the specific wound condition and sutures were removed on 14 days post-operatively as part of the post-operative management protocol.

### Control group

Cold compression was applied to all dogs for 15 min, 2–3 times a day, on the 1^st^ day after surgery and continued for 3 days. All dogs received exercise at home performed by the owners, including PROM 10–15 times and standing exercise for 1–3 min, repeated 3 times and with a rest for 1 min during each set (days 7–84), warm compression for 15 min and PROM 10–15 times, twice a day (days 14–84), and daily leash walking for 5–10 min (days 21–84).

### ES and LASER groups

All dogs in both treatment groups visited the rehabilitation unit to undergo aquatic exercise using an underwater treadmill in addition to the control group. The exercise regimen, including speed and duration, was individually customized for each dog. These sessions started on day 14 and continued until the end of the study. Two self-adhesive electrodes (size 3.2 cm, round) were attached to the medial and lateral sides of the stifle joint in the ES group. The electrical stimulator was configured for IFC with a carrier frequency of 4000 Hz, AMF of 100 Hz, pulse duration of 250 μs, and phase duration of 125 μs on days 7, 14, 21, 28, 35, 42, 49, 56, 70, and 84 following surgery. LASER was administered at a dosage of 4–8 J/cm^2^ on days 7, 14, 21, 28, 35, 42, 49, 56, 70, and 84 post-operatively in the LASER therapy group.

### Evaluation parameters

Lameness score, pain score, thigh muscle circumference (TMC), and ROM of the stifle joint were assessed and recorded by the same veterinarian before surgery and during each post-operative visit until the end of the study. Lameness and pain scores were determined on a scale of 0–5 and 0–4, respectively. Both lameness and pain scores were used as changes in the scoring. TMC was measured at 70% of the femoral length from the greater trochanter to the lateral fabella in lateral recumbency using a Gulick measuring tape. The ROM of the stifle joint was measured with a goniometer at the angles of maximal flexion and extension, ensuring the comfort of the dogs during the measurements. Measurements of both TMC and ROM are expressed as percentage changes. To calculate the average values for each dog, three measurements of TMC and ROM were taken. These evaluations were assessed before surgery and on 1, 7, 14, 21, 28, 42, 56, 70, and 84 days post-operatively.

In addition, owners evaluated the canine brief pain inventory (CBPI) according to an explanation from a veterinarian. The CBPI questionnaire encompassed three components: Pain severity score (PSS), pain interference score (PIS), and QOL evaluation [20, 21]. PSS and PIS were determined on the basis of responses to four questions and six questions, respectively. Each PSS and PIS question was rated on a 10-point scale, resulting in maximum scores of 40 and 60 points, respectively. QOL was rated on a five-category scale: 1 = poor, 2 = fair, 3 = good, 4 = very good, and 5 = excellent. CBPI questionnaires were assessed before surgery and postoperatively on days 7, 14, 21, 28, 35, 42, 49, 56, 70, and 84.

### Statistical analysis

The Chi-square test for sample demographics and surgical procedures was used to assess the homogeneity of the treatment groups. A two-way mixed model analysis of variance followed by Tukey’s multiple comparison or Kruskal–Wallis tests followed by Dunn’s multiple comparison test, depending on the distribution of the data, was used to compare the evaluation parameters among groups at each visit. Data are presented as mean ± standard deviation (SD) or median with interquartile range. p < 0.05 was considered statistically significant. Data were analyzed using GraphPad Prism software (GraphPad Software Inc., La Jolla, CA, USA).

## Results

A total of 26 client-owned dogs comprising 31 stifles were enrolled in the study (Table-1). Pomeranians (n = 10), chihuahuas (n = 13), Yorkshire Terriers (n = 1), and mixed breeds (n = 2) were included in the study. Five Pomeranians underwent bilateral MPL surgery during separate sessions. The control and ES groups each consisted of 10 stifles, whereas the LASER group consisted of 11 stifles. The mean ± SD age and weight of all dogs were 34.97 ± 30.18 months and 3.34 ± 0.99 kg, respectively. A statistically significant difference in age was observed between the control and LASER groups (p *=* 0.039), whereas no significant differences were identified in body weight, body condition score, sex, or breed. Pre-operative evaluation of lameness and pain scores in all dogs yielded median values of 1 (1–2) and 0 (0–1), respectively. Significant differences were observed between the groups, with the lameness score in the LASER group being higher than that in both the control and ES groups (p *=* 0.008). In addition, there was a significant difference in pain scores between the ES and LASER groups (p *=* 0.039).

**Table-1:** Demographic variables, affected limb, patellar grading, lameness score and pain score at the pre-operative assessment (day 0), along with the surgical technique used in the control, ES, and LASER groups.

Variable	Control	ES	LASER	p-value
Number of stifles	10	10	11	
Age (month, mean ± SD)	22.6 ± 19.1^b^	27.5 ± 27.4	53.0 ± 34.1^b^	0.039*
Body weight (kg, mean ± SD)	3.51 ± 1.00	3.29 ± 0.81	3.23 ± 1.19	0.812
Body condition score (mean ± SD)	3.00 ± 0.67	3.40 ± 0.70	3.27 ± 0.47	0.344
Affected limb				0.446
Right	8	6	6	
Left	2	4	5	
Medial patellar luxarion grade				0.105
2/4	10	6	5	
3/4	0	3	4	
4/4	0	1	2	
Lameness score (median ± IQR)	1.00 (1.00–1.00)^b^	1.00 (0.25–1.75)^c^	2.00 (1.50–3.00)^b,c^	0.008*
Pain score (median ± IQR)	0.50 (0.00–1.00)	0.00 (0.00–0.00)^c^	1.00 (0.50–1.50)^c^	0.039*
Surgical procedure				0.653
Lateral imbrication	10	10	11	
Medial desmotomy	2	6	7	
Trochlear block recession	10	10	11	
Tibial tuberosity transposition	1	5	5	

^a^significant difference was observed between control and ES group

b significant difference was observed between control and LASER group

c significant difference was observed between ES and LASER group. IQR=Interquartile range, ES=Electrical stimulation, LASER=Light amplification by stimulated emission of radiation

Among the 31 stifles, the right limb was affected in 65% (20/31) of cases and the left limb in 35% (11/31) of cases. The distribution of MPL grading was as follows: 68% (21/31), 22% (7/31), and 10% (3/31) of patients had grade 2 MPL, grade 3, and grade 4, respectively. No statistically significant differences were observed in the affected limb or MPL grading among the three groups. With regard to the surgical procedures used to correct MPL, lateral imbrication and TBR techniques were applied in all dogs (100%, 31/31); medial desmotomy was used in 48% (15/31) and TTT in 35% (11/31). No significant differences between the groups were observed in the surgical techniques used for MPL correction.

**Figure-1:**
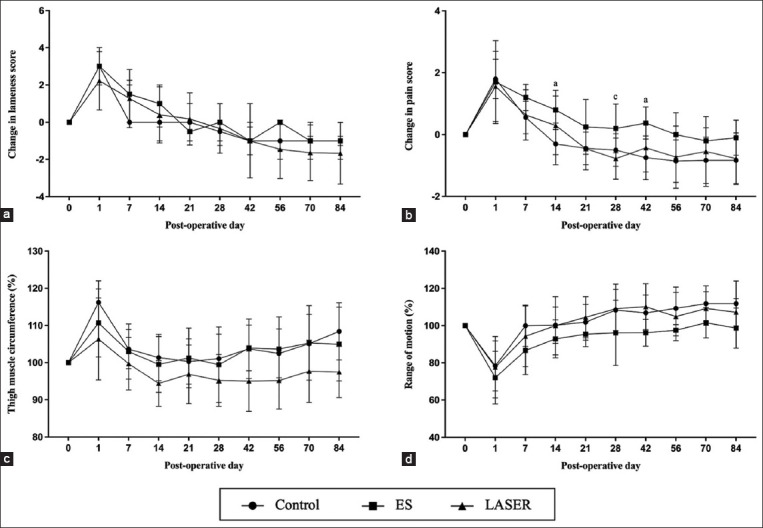
(a) Change in lameness score, (b) change in pain score, (c) thigh muscle circumference (%), and (d) range of motion (%) at the pre-operative assessment (day 0), along with the post-operative follow-up day 1, 7, 14, 21, 28, 42, 56, 70, and 84 in the control, electrical stimulation (ES) and light amplification by stimulated emission of radiation (LASER) groups. Data represented as mean ± standard deviation (SD). (^a^significant difference was observed between control and ES group, ^b^significant difference was observed between control and LASER group, ^c^significant difference was observed between ES and LASER group).

Post-operative changes in lameness and pain scores showed similar trends in all groups. Changes in lameness and pain scores significantly increased in all groups on day 1 following surgery, followed by a gradual decrease (Figures-1a and b). However, there was a slower decrease in pain scores in the ES group than in the other groups. The results revealed a statistically significant difference in pain scores between the ES group and the control and LASER groups. Differences between the ES and control groups were evident on days 14 (p = 0.011) and 42 (p = 0.016). Differences were observed on day 28 (p = 0.017) post-operatively compared to the LASER group.

No significant differences were observed in either TMC or ROM among any group. Following the surgical correction of MPL, there was a notable increase in TMC, reaching a peak on post-operative day 1, ranging between 106% and 116% compared to pre-operative measurements. Throughout the follow-up period, TMC in all groups exhibited a similar trend and gradually decreased over time. At the conclusion of the study, TMC ranged from 97% to 108% compared with pre-operative TMC values. Although the LASER group showed the lowest measured TMC, no significant differences were detected between the other groups (Figure-1c). The most significant decrease in ROM was observed on day 1 after surgery, with a reduction of up to 28% compared to the pre-operative ROM. At the end of the study, ROM ranged between 97% and 112% compared to pre-operative ROM. Throughout the study, ROM in all groups followed a similar trend, with no significant differences observed among the groups at any time point (Figure-1d). Furthermore, despite significant differences in both TMC and ROM between pre-operative and post-operative day 1, these parameters returned to baseline values by post-operative day 7 after surgery.

**Figure-2:**
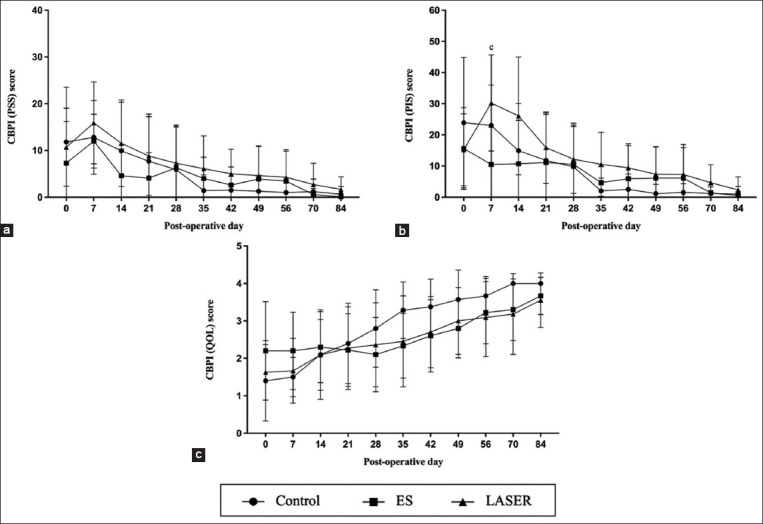
(a) The pain severity score (PSS), (b) pain interference score (PIS), and (c) the dog’s quality of life (QOL) from canine brief pain inventory (CBPI) questionnaires at the pre-operative evaluation (day 0), along with the post-operative follow-up day 7, 14, 21, 28, 35, 42, 49, 56, 70, and 84 in the control, electrical stimulation (ES) and light amplification by stimulated emission of radiation (LASER) groups. Data represented as mean ± standard deviation (SD). (^a^significant difference was observed between control and ES group, ^b^significant difference was observed between control and LASER group, ^c^significant difference was observed between ES and LASER group).

The CBPI scores for PSS and PIS showed consistent trends, indicating a gradual decrease from the pre-operative assessment throughout the study period. Notably, no significant differences were observed in PSS scores among any groups (Figure-2a). However, a significant increase in the PIS score was observed in the LASER group compared with the ES group at a specific time point, particularly on post-operative day 7 (p = 0.019) (Figure-2b). Concerning the CBPI score for QOL, our initial observations, conducted before the surgical correction of MPL, revealed that most owners perceived their dogs’ QOL to be predominantly poor to fair. By the end of the study, however, a significant shift occurred, with most owners reporting a gradual improvement in their dogs’ QOL, which resulted in ratings of good to very good (Figure-2c).

## Discussion

In recent years, there has been a growing interest in companion animal rehabilitation in the field of veterinary medicine [8, 11, 22]. This study focuses on both primary rehabilitation management and post-operative rehabilitation approaches [9, 14, 23, 24]. In primary management, the emphasis of rehabilitation is on enhancing the animal’s physical functionality and alleviating any associated pain [12]. This role becomes particularly important in situations where surgical interventions may not be the most suitable option, often due to the animal’s specific condition or the owner’s preferences. Primary rehabilitation plays a crucial role in enhancing the overall QOL of these animals [11, 25, 26] as a conservative treatment approach. However, post-operative rehabilitation may not generally be mandated for every surgical procedure or animal [9]. Although some surgical interventions effectively address the primary issue, they may not completely restore the animal’s previous levels of physical activity. This can be attributed to factors such as concurrent soft tissue trauma, the potential development of osteoarthritis, or long-term disuse of the affected limb, all of which may prevent complete recovery of the patient. As a result, post-operative rehabilitation therapies have emerged as a crucial aspect of post-operative care, aiming to facilitate recovery and enhance the overall well-being of the animal [8, 10, 27]. However, our study demonstrated that there were no significant differences in the improvements between dogs that underwent post-operative rehabilitation by a veterinarian and those that only underwent home exercises prescribed by the owner. In addition, there were no significant differences in the efficacy of ES and LASER therapy.

This study revealed that changes in post-operative lameness and pain scores evaluated on day 1 after surgery were highly increased in all groups, which was expected and was associated with typical post-operative pain and inflammation. It is noteworthy; however, that post-operative pain and inflammation were effectively resolved within 7–14 days after surgery. This finding agrees with a previous study by Morton *et al*. [28] that post-operative pain can persist for 10–14 days following most routine soft-tissue surgeries. Although our MPL correction surgery involved reconstruction of both bone and soft tissue, it is important to note that the degree of bone involvement was comparatively less than that in other orthopedic surgeries. If osteotomy techniques are used, the pain may be much more intense and prolonged than that observed in this study.

In the present study, we expressed both lameness and pain scores, as well as TMC and ROM measurements, as changes in scoring or percentage changes rather than using absolute measurements or measurement units. This approach has been adopted to eliminate individual differences, including breed, dog size, or any other physical differences, and to allow for a more uniform comparison. Regarding the change in lameness score, no significant differences were observed among the various post-operative management methods. At the end of the study, particularly on day 84 post-operatively, the lameness score appeared to be equal to or even lower than the pre-operative score. We observed a slower decrease in the pain score in the ES group than in the control and LASER groups. This significant difference was primarily observed before the initiation of post-operative rehabilitation and at certain points after rehabilitation. Throughout the study period, the decrease in the pain score in the ES group was consistently lower than that in the other groups. We suggest that this observed trend in the ES group may be attributed to the group’s initially lower pain score at the pre-operative evaluation. In addition, the comparison in this study used the change in pain score, which could make the decrease in pain score seem slower than in the other groups due to the lower pain score at the beginning of the study.

TMC measurement was used to assess limb muscle mass, reflecting the extent of limb usage and muscle development [29, 30]. A higher TMC indicates a greater muscle mass, indicating a limb in better condition. Our results showed an increase in TMC in all groups following surgery. This increase was mainly due to inflammation, which resulted in swelling and edema of the limbs, rather than a real increase in muscle mass. Post-operative swelling of the limb gradually resolved by day 7 after surgery. This resolution is likely due to a combination of the body’s self-resolving inflammatory response, anti-inflammatory medication, and cold compression administered by the owner [31]. A decrease in ROM was observed in all groups following surgery, indicating a reduction in physiological movements. Reduction in ROM was directly associated with post-operative factors, including inflammation, swelling, and pain. An inverse relationship was observed between TMC and ROM on day 1 after surgery. Thus, ROM increased as inflammation subsided, and this improvement was observable by day 7 after surgery.

TMC and ROM showed no significant differences between groups throughout the follow-up period. Interestingly, both post-operative rehabilitation groups, which included ES and LASER therapy along with underwater treadmill exercises, showed no significant advantage compared with the control group that relied solely on home exercise. Considering the generally beneficial effects of underwater treadmill exercises [8, 32, 33], this outcome was somewhat unexpected. Previous studies have indicated that introducing underwater treadmill exercises as early as 2–4 weeks post-operatively can be advantageous in orthopedic surgeries, such as CrCLR repair. This approach promotes weight-bearing, enhances early active ROM, and facilitates muscle strengthening [8, 32, 33]. In addition, we believe that such an approach would be highly beneficial in the context of MPL surgery. However, the lack of a significant difference between the control group and the two post-operative rehabilitation groups may be related to the characteristics of MPL correction surgery. Typically, dogs that undergo MPL correction spontaneously regain normal limb function within 1 week of surgery. This early return to limb function may minimize the loss of muscle mass, which is often observed in other orthopedic cases. Therefore, we propose that there may be no requirement for additional rehabilitation programs, such as underwater treadmill exercises [8], if owners consistently and appropriately perform home exercises.

In addition, the evaluation from the owner’s perspective through the CBPI score revealed no significant differences among the groups after day 7, which marked the initiation of the rehabilitation program. The gradual reduction in both PSS and PIS indicated a decrease in the perceived post-surgical pain. Consequently, it improved the overall QOL of the dogs, a trend supported by the CBPI scoring. Our CBPI results underscore that the pain resulting from MPL correction surgery is transient and the dogs experience an enhanced QOL following the correction of this condition. These findings suggest that while MPL may not pose a life-threatening risk, it can significantly affect the overall well-being and comfort of dogs [4, 7]. These suggestions further supported the reduction in lameness scores across all groups after the surgery. Therefore, we strongly recommend surgical correction of MPL in dogs because it represents an abnormal condition that can potentially impact QOL [1, 2, 4, 34].

Despite their differing mechanisms of action, no differences were observed between ES and LASER therapy. Interestingly, neither ES nor LASER therapy resulted in greater recovery after MPL surgery, which was contrary to our expectations, as we believed that the analgesic effects of these approaches would promote a more rapid post-operative recovery [12]. Previous studies revealed that ES pain control mechanisms involve stimulating signals from A-β fibers to activate inhibitory neurons in the substantia gelatinosa of the dorsal horn of the spinal cord [15–17]. This action prevents the transmission of pain impulses from the peripheral region to the brain. Conversely, LASER therapy may influence pain perception through direct or indirect actions on nociceptors, as well as modulation of inflammation, which can contribute to pain relief [14, 18, 19]. We hypothesized that the similar outcomes in all groups, regardless of treatment differences, were due to the moderate and non-persistent nature of pain following MPL correction that did not involve osteotomy techniques. This pain can be effectively controlled by NSAIDs alone, which are prescribed in all groups. It is worth considering that if the surgery resulted in severe and persistent pain, necessitating multimodal analgesia, or if the use of NSAIDs post-operatively had been restricted, or if ES and LASER therapy had been initiated earlier and more frequently, the differences in outcomes between the treatment groups might have been more obvious.

This study has some limitations. First, the relatively small sample size may have affected the statistical power of this study. Second, the majority of the study’s sample had MPL grade 2, which typically presents with mild lameness and pain. As a result, the surgical correction was relatively less invasive, leading to rapid recovery. We recommend that further studies with a larger representation of different MPL grades would provide a better understanding of the benefits of rehabilitation.

## Conclusion

Post-operative rehabilitation following MPL correction surgery may promote limb usage, enhance joint function, increase muscle mass, and relieve pain. However, the duration and level of pain experienced post-operatively may influence the necessity for rehabilitation. Our findings indicate that if these exercises are consistently and correctly performed, there is no significant difference in recovery outcomes between dogs receiving veterinarian-supervised post-operative rehabilitation and those solely engaged in home exercises. Moreover, ES and LASER therapy offer similar pain-relieving effects following MPL surgery; therefore, the choice between ES and LASER therapy depends on the availability of equipment and veterinarian preference.

## Authors’ Contributions

EA, IK, and CW: Designed the study and interpreted the data. EA and IK: Statistically analyzed the results. CM, PS, SA, OM, AT, and CW: Conducted the study and collected the data. EA: Wrote the manuscript. CW: Supervised the study. All authors have read, reviewed, and approved the final version of the manuscript.
